# Primary Renal Epithelioid Hemangioendothelioma

**DOI:** 10.1155/2012/802515

**Published:** 2012-10-24

**Authors:** S. Roy, A. V. Parwani

**Affiliations:** Department of Pathology, University of Pittsburgh Medical Center, 3550 Terrace Street, A614 Scaife Hall, Pittsburgh, PA 15261, USA

## Abstract

Epithelioid hemangioendothelioma is a rare vascular tumor with intermediate biologic behavior and metastatic potential. Primary renal epithelioid hemangioendothelioma is extremely rare and we present the second report of this rare tumor in an interesting clinical scenario. A 59-year-old male with established history of widely metastatic high grade esophageal adenocarcinoma was found to have an isolated renal nodule on a followup computed tomography (CT) scan. Surgical excision, with the suspicion of metastatic carcinoma, and subsequent pathologic examination revealed an epithelioid hemangioendothelioma. The various differential diagnoses and use of morphological clues and immunohistochemistry are discussed.

## 1. Introduction

Epithelioid hemangioendothelioma is a rare angiocentric vascular tumor of intermediate biologic behavior with metastatic potential. It was initially grouped along with other hemangioendotheliomas—hobnail and kaposiform; however due to the aggressive biological nature, it is now grouped with angiosarcomas in the current WHO classification [1]. Hemangioendotheliomas have been reported in various sites; most commonly in soft tissues followed by liver, lung, bone, peritoneum, stomach, thyroid, and central nervous system [2–6]. Kidney is an extremely rare site, especially for the epithelioid variant. There are very few case reports documenting hemangioendothelioma in the kidney [2, 6–8]. Karasavvidou et al. [2] described the first and only case of primary renal epithelioid hemangioendothelioma in a 54-year-old woman.

## 2. Case Report

**Figure 1:**
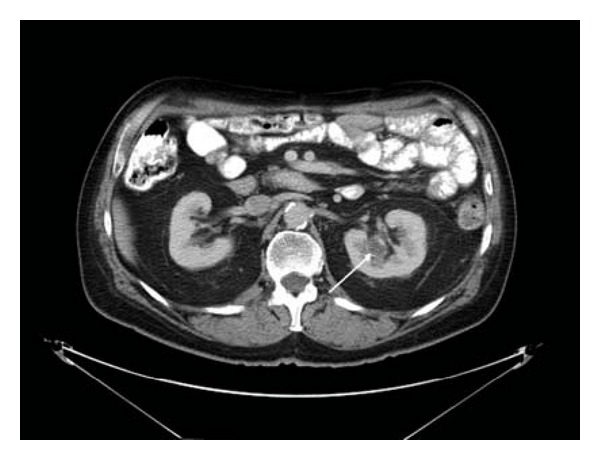
CT scan of the abdomen showing a 2.2 cm solid lesion in the midpole of the left kidney near the pelvis (white arrow).

A 59-year old hypertensive and diabetic male, with a past medical history significant for a T3N1 M1, high grade esophageal adenocarcinoma, status post-esophagectomy with subsequent right frontal lobe metastasis, and status post-stereotactic radiosurgery, presented for routine followup. He was on surveillance to monitor disease progression for the past 2 years, when a 2.2 cm solid lesion was noted in the mid pole of the left kidney near the pelvis on computed tomography (CT) (Figure 1). His renal function tests were within normal limits and a cystoscopic evaluation of his collection duct system revealed no filling defects. Due to prior history of malignancy, the renal lesion was highly suspicious for metastasis and a partial nephrectomy was performed. Intraoperatively a small, soft, and cystic mass was identified which was completely enucleated and sent for pathologic examination.

**Figure 2:**
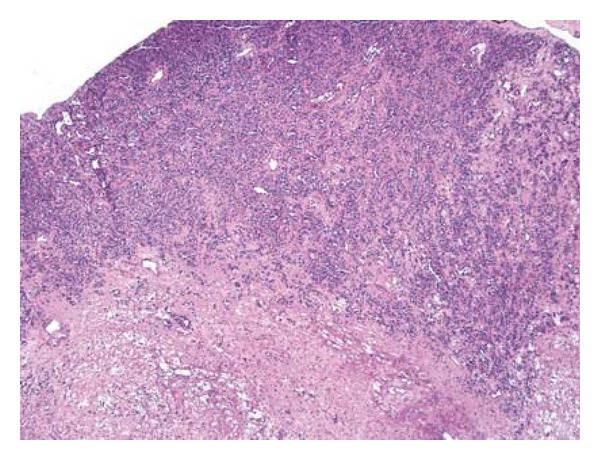
Low-power view of the cellular tumor forming slit-like vascular spaces, cords, and small nests. The central area is relatively hypocellular and edematous (hematoxylin & eosin, low power).

**Figure 3:**
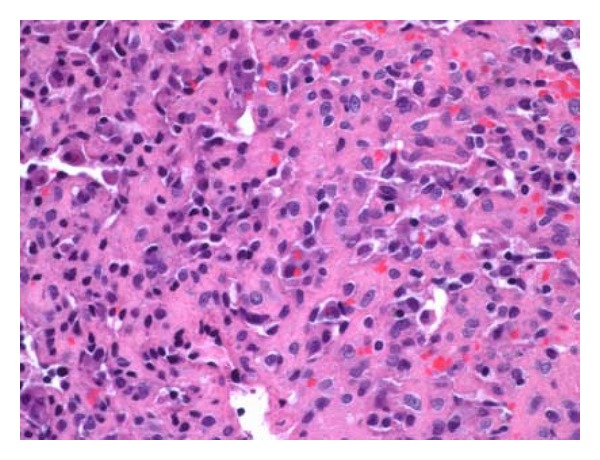
High-power view shows epithelioid tumor cells with mild to moderate nuclear pleomorphism, moderate amount of eosinophilic cytoplasm, and lining slit-like vascular spaces. Occasional tumor cells show intracytoplasmic lumen and red blood cells. No mitoses or necrosis was identified in the entire tumor (hematoxylin and eosin, high power).

**Figure 4:**
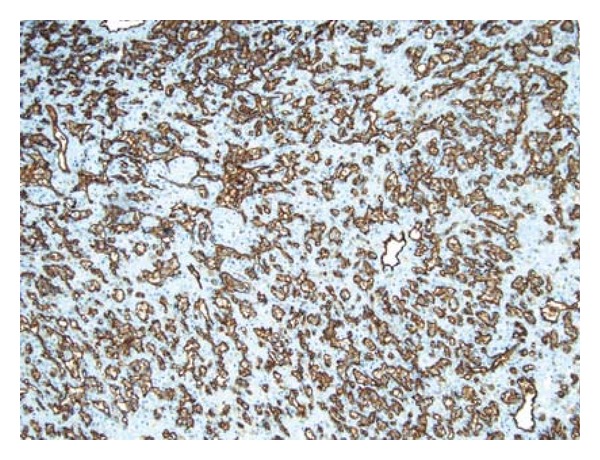
A CD31 immunostain clearly highlights the vascular spaces and the lining tumor cells (DAB chromogen, medium power).

**Figure 5:**
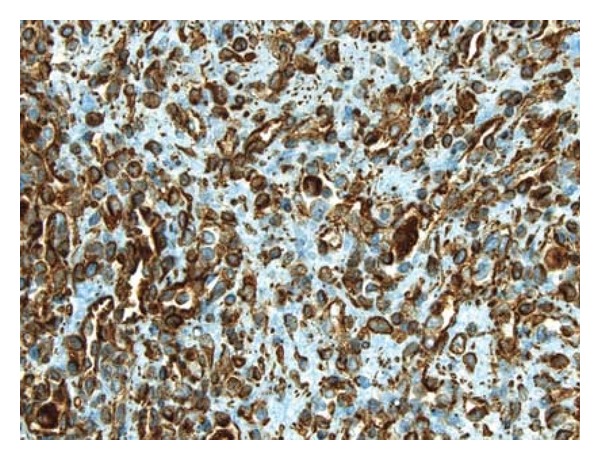
Vimentin immunostain highlights most of the tumor cells strongly (DAB chromogen, high power).

Gross examination revealed an irregular tan-pink soft tissue with no apparent renal parenchyma. The histologic section demonstrated an ill-defined, dense cellular infiltrate with irregular slit-like spaces, cords, and nests, lined by the atypical tumor cells, containing few red blood cells (Figure 2). The tumor cells had moderate amount of eosinophilic cytoplasm, single hyperchromatic nucleus, and inconspicuous nucleolus with mild to moderate pleomorphism. Occasional cells demonstrated abundant cytoplasm with intracytoplasmic lumen, containing red blood cells (Figure 3). No mitoses or areas of necrosis were identified. The stroma was sclerotic to focally myxoid in character. Immunohistochemical studies showed expression of CD31 (Mouse monoclonal, DAKO) (Figure 4) and Vimentin (Mouse monoclonal, Ventana) (Figure 5) and negativity for pancytokeratin (mouse monoclonal, DAKO) and HMB-45 (monoclonal mouse, ventana) in the tumor cells. The overall histologic and immunohistochemical features were diagnostic of epithelioid hemangioendothelioma. Microscopic examination of the entire specimen did not reveal any normal renal parenchyma.

## 3. Discussion

Epithelioid hemangioendothelioma is a rare angiocentric tumor of intermediate biological behavior. Epithelioid variant of hemangioendothelioma is particularly more aggressive than the other variants [1, 3]. This has led to its inclusion under angiosarcomas in the current WHO classification [1]. This tumor has been reported in various sites; however, to the best of our knowledge, there is only one well-documented case of primary epithelioid hemangioendothelioma of kidney in the literature to date [2]. Epithelioid hemangioendothelioma tends to occur over a wide range of age groups excluding childhood and affects both sexes equally [1–3].

Histological examination is remarkable for cellular tumor growth in shorts strands, cords, and solid nests in a sclerotic to myxoid stroma. The cells have moderate to abundant amount of eosinophilic cytoplasm with intracytoplasmic vacuoles containing red blood cells, recapitulating primitive vascular lumens. The latter is a characteristic feature of this tumor. The cells appear bland with rare mitoses [1–3]. The histological features described by Karasavvidou et al. [2] are similar to the findings in our case. Histologic features that confer aggressive behavior include nuclear atypia, coagulative necrosis, mitosis >1/10 HPF, and excessive spindling of tumor cells [1–3]. These features were not seen in our case.

Immunohistochemically, expression of CD31, CD34, and FLI-1 is the most reliable feature for diagnosis of this tumor [1, 2]. In our case the tumor cells expressed CD31 and were negative for pancytokeratin which ruled out a possibility of a metastatic adenocarcinoma.

Epithelioid hemangioendothelioma is a great mimicker of other tumors including carcinomas, sarcomas and melanomas, primarily due to the presence of “epithelioid” cells. Use of limited immunohistochemistry utilizing endothelial markers is helpful in making a definitive diagnosis [2, 3]. In our case it was especially helpful as the patient already harbored a high grade adenocarcinoma with known metastasis and the renal nodule was clinically highly suspicious for metastasis. We also performed HMB-45 stain which was negative and this helped in ruling out angiomyolipoma, which also has morphological features overlapping with epithelioid hemangioendothelioma.

Surgical excision of the lesion confers accurate diagnosis and definitive treatment [2]. Nephron sparing surgery is the treatment of choice and has been widely accepted for this lesion. This is especially true for lesions up to 4 cm in size and have longterm survival similar to patients offered radical nephrectomy with an added advantage of preserving renal function [2, 9, 10]. Also with the use of this surgical technique, frozen section is usually not necessary since the kidney is preserved. However does not always hold true for larger sized tumors [2, 9].

The disease related mortality of epithelioid hemangioendothelioma varies depending on anatomic sites. It is approximately 13% with soft tissue lesion in contrast to 65% and 35% with lung and liver lesions, respectively [3]. Survival data for renal lesion is unclear due to extremely few documented cases.

In conclusion it is important to accurately diagnose epithelioid hemangioendothelioma in kidney as it is a rare entity and mimics other tumors, especially carcinomas. Situations like in our case can generate further diagnostic confusion and it is important to be aware of the existence of this lesion at this site, as treatment approaches differ significantly.

## References

[1] Fletcher CDM, Unni KK, Mertens F (2002). *Pathology and Genetics of Tumours of Soft Tissue and Bone*.

[2] Karasavvidou F, Barbanis S, Gravas S (2009). Primary renal epithelioid hemangioendothelioma. *Onkologie*.

[3] Weiss SW, Goldblum JR (2008). Hemangioendothelioma. *Soft Tissue Tumors*.

[4] Sanjay P, Raman S, Shannon J, Williams GT, Woodward A (2005). Gastric epithelioid haemangioendothelioma: a rare cause of upper gastrointestinal bleeding. *Postgraduate Medical Journal*.

[5] Mehrabi A, Kashfi A, Fonouni H (2006). Primary malignant hepatic epithelioid hemangioendothelioma: a comprehensive review of the literature with emphasis on the surgical therapy. *Cancer*.

[6] Celikel C, Yumuk PF, Basaran G, Yildizeli B, Kodalli N, Ahiskali R (2007). Epithelioid hemangioendothelioma with multiple organ involvement: report of two cases and review of the literature. *Acta Pathologica, Microbiologica et Immunologica Scandinavica*.

[7] Chatterjee D, Powell A (1982). Renal hemangioendothelioma. *International Surgery*.

[8] Gonzalez-Crussi F, Sotelo-Avila C, Kidd JM (1981). Mesenchymal renal tumors in infancy: a reappraisal. *Human Pathology*.

[9] Ljungberg B, Hanbury DC, Kuczyk MA (2007). Renal cell carcinoma guideline. *European Urology*.

[10] Russo P (2007). For small renal tumors, kidney sparing surgery provides local tumor control and prevents chronic kidney disease. *Onkologie*.

